# Ethnic identity and resilience: a moderated mediation analysis of protective factors for self-blame and racial microaggressions

**DOI:** 10.3389/fpsyg.2023.1198375

**Published:** 2023-06-29

**Authors:** Aldo M. Barrita, Gloria Wong-Padoongpatt

**Affiliations:** Psychological and Brain Sciences, Department of Psychology, University of Nevada, Las Vegas, Las Vegas, NV, United States

**Keywords:** racial microaggressions, self-blame, psychological distress, ethnic identity, resilience

## Abstract

**Introduction:**

People of Color (PoC) in the United States encounter everyday racial microaggressions, and these commonplace experiences can wear and exhaust PoC’s resources. Racial microaggressions have shown detrimental effects on physical and psychological well-being. Consequently, researchers have examined and tested different ways in which PoC cope and protect themselves from these everyday exchanges. Past findings have indicated that PoC might blame themselves for racism-related occurrences to cope with these commonplace discriminatory experiences. Ethnic identity and resilience have emerged in research as protective factors that can moderate and buffer the impact of racism on PoC’s well-being. We used a combination of mediation, moderation, and conditional analyses to unpack the relationships between racial microaggression (predictor), psychological distress (outcome), self-blame (mediator), resilience (moderator), and ethnic identity (moderator).

**Methods:**

This study used a cross-sectional design and sampled 696 PoC regarding their experiences and responses to racial microaggressions. We tested the association between psychological distress and racial microaggressions and further examined whether self-blame mediated the relationship. We also tested ethnic identity and resilience as moderators and used a conditional analysis to determine whether these protective factors moderated the mediation model.

**Results:**

Findings from the mediation, moderation, and conditional analyses supported our four hypotheses: (H1) self-blame mediated the relation between racial microaggressions and psychological distress (mediation), (H2) ethnic identity moderated the association between racial microaggressions and self-blame but only at low and average levels (moderation), (H3) resilience moderated the relation between self-blame and psychological distress but only at low and average levels (moderation), and (H4) evidence of moderated mediation were found for all five variables (conditional). While statistically significant, most moderation effects were minimal to small.

**Conclusion:**

PoC may engage in self-blame when experiencing racial microaggressions, which explains why these everyday, commonplace occurrences might lead to psychological distress. There was evidence that ethnic identity and resilience can protect PoC from the negative effects of racial microaggressions. These buffering effects, however, only emerged for PoC endorsing high levels of ethnic identity and resilience, and it should be noted that for most participants, the link between racial microaggressions and psychological distress was still significant. Future studies might need to explore additional individual and interpersonal alongside institutional factors that can protect PoC from racism-related harms.

## Introduction

People of Color (PoC) in the United States (U.S.) have experienced centuries of racism (both interpersonal and institutional). Racial oppression in the U.S. continues in cultural practices, commonplace social exchanges, and institutional policies (Crenshaw, 1990). Racial tension more recently has heightened in the U.S., with White and PoC reporting an increase in race-related struggles (Pew Research Center, 2019). The violence of racism in the U.S. has experienced many ebbs and flows—from blatant and attention-grabbing to covert and less visible. The consensus from decades of research findings has indicated that repeated experiences with racism can have a detrimental impact on the physical and psychological well-being of PoC (American Psychological Association, 2016). Thus, researchers have emphasized the protective factors for PoC confronting discrimination practices, power dynamics, and oppressive environments (Suyemoto et al., 2022). Scholarship on racism (Pieterse and Powell, 2016) have explored the effects of different societal levels (e.g., individual, interpersonal, and institutional), and findings do suggest that PoC may be self-blaming and internalizing the racism-related sentiments toward their racial groups (Clark and Clark, 1947; Helms, 1990; David, 2013; Wong-Padoongpatt et al., 2022b). Less is known, however, about this tendency of PoC to self-blame as it relates to more hidden forms of racism, such as racial microaggressions (Sue et al., 2007; Wong et al., 2014). Researchers have explored some internal processes at the individual level, such as ethnic identity and resilience, as protective factors against the effects of racism (Brown and Tylka, 2011; Nissim, 2014). The bulk of the literature has highlighted the beneficial aspects of ethnic identity and resilience as empowering traits for PoC, particularly when experiencing commonplace racism, such as racial microaggressions (Barrita and Wong-Padoongpatt, 2021; Sims-Schouten and Gilbert, 2022).

Racial microaggressions are everyday slights, insults, and indignities targeting PoC (Sue et al., 2007) occurring at the microsystem level (Bronfenbrenner, 1992; Sue et al., 2019). There is evidence that PoC experience psychological harm from racial microaggressions (Wong-Padoongpatt et al., 2017; Sue et al., 2019; Williams, 2020; Barrita, 2021; Cheng et al., 2021). For example, Abreu et al. (2023) found that intersectional microaggressions (racial and sexual) were associated with symptoms of depression among Latinx LGBTQ+ youth. In a series of experimental studies, Wong-Padoongpatt et al. (2017) found that manipulating microaggressions decreased implicit self-esteem and increased physiological stress among Asian American participants (Wong-Padoongpatt et al., 2020). Recent findings have also revealed that PoC can use negative or harmful strategies when coping with racial microaggressions (Polanco-Roman et al., 2016). In a diverse PoC sample, Barrita et al. (2023a) found that experiences with racial microaggressions were closely associated with substance use coping, and this effect was further mediated by psychological distress. Overall, racial microaggressions appear to elicit various types of undue harm to PoC.

Other maladaptive coping strategies, such as internalized racism, have been explored (see David et al., 2019, for a review). A few studies have examined in more detail the associations between racial microaggressions, internalized racism, and mental health. Wong-Padoongpatt et al. (2022a,b,c) recently addressed these relations in a series of studies examining the lived experiences of Asian Americans during the COVID-19 pandemic. The overall message was that Asian Americans not only experienced more everyday, commonplace racism but also reported higher internalized racism compared to other racial groups during the pandemic. According to the Internalized Oppression Theory (IOT; David, 2013, p. 14), people who are oppressed are likely to endorse self-defeating and negative cognitions, attitudes, and behaviors.

Self-blame is an aspect of internalized racism that PoC may use as a maladaptive coping strategy. That is, PoC may cope with racism by blaming themselves for these occurrences. Researchers have found links between self-blame and poor mental health among different communities of color (Wei et al., 2010; Szymanski and Lewis, 2016; Lei et al., 2022). Szymanski and Lewis (2016) found that self-blaming and detachment coping strategies were mechanisms for the effect of gendered racism on psychological distress among African American women. Similarly, among Asian Americans, Lei et al. (2022) found that self-blame coping strategies predicted distress. Moreover, when distress was associated with racism, Lei et al. (2022) found that self-blame mediated this relationship. A recent study (Barrita et al., 2023b) explored racial microaggressions specific to immigration using a sample of Asian and Latinx college students and found evidence of self-blame coping strategies. Specifically, during xenophobic attacks, Asian and Latinx students were more likely to engage in self-blame coping strategies associated with negative mental health effects. Similarly, other studies have explored self-blame and racial microaggressions using diverse PoC college samples (Wong-Padoongpatt et al., 2022b) or samples with multiple marginalized identities, such as Queer PoC (Barrita et al., 2023c). Since self-blame can be an integral part of racism-related experiences, the current study also tested self-blame as a mediator for racial microaggressions and the connection to psychological distress (Sue et al., 2019; Williams et al., 2020).

Before discussing possible protective factors for racism, particularly ethnic identity, it is important to highlight specific differences between the terms *race* and *ethnicity,* given that the current study explores both when it comes to PoC’s experiences with racial microaggressions. Race is defined here as a social and cultural construct that categorizes and separates groups based primarily on physical traits such as the color of skin, hair, and eyes (Bandura and Walters, 1977; Chang et al., 2023). Historically, PoC and other groups (e.g., Jews, Irish people) in the U.S. have been *racialized* based on White supremacy ideologies where Whiteness is upheld as the standard; and therefore, White people are positioned to acquire social dominance (Bandura and Walters, 1977; Helms, 1990). The process of *racialization* carries social, economic, and political factors that reinforce a system of racial oppression against those seen as less or non-White (Fredrickson, 2002) and gives power and privilege to White people (Helms, 1990). Racial microaggression is one example of racialization occurring in everyday exchanges. U.S. federal practices, such as the nationwide census, have historically changed and redefined racial categories. Hyphenated racial identities (e.g., Asian-American) in official federal documents can also be perceived as a form of division from White people. A person’s racial group membership is socially imposed, leaving both PoC and White people little agency to choose their race (Bandura and Walters, 1977; Helms, 1990). For White people, the process of acknowledging their racial identity exists in the context of racism and requires recognizing their racial power and privilege (Helms, 1990).

Ethnicity, on the other hand, is a fluid, non-exclusive cultural construct (Phinney, 1992) that incorporates heritage, genetic backgrounds (e.g., Samoan, Irish), and even nationality (e.g., Mexican, Spanish). Compared to race, ethnicity does not aim to divide; instead, it makes room for overlapping identities. Race and ethnicity are sometimes mistakenly treated as the same in the literature, therefore erasing identities within groups with a history of diversity or colonization (e.g., Afro-Latinx, Indigenous Latinx, Black Asians; Adames et al., 2021). One of the aspects in which ethnicity can be assessed is ethnic identity. And as mentioned, ethnic identity has been described as a protective factor for racism in the literature (Brown and Tylka, 2011; Nissim, 2014). Ethnic identity can be defined as culturally and developmentally informed beliefs, thoughts, and self-perceptions about ethnicity’s meaning, value, and significance (Phinney, 1992; Swanson et al., 2009). The development of ethnic identity for PoC is partially informed by overcoming social rejection (Helms, 1990; Phinney, 1992; Jones and Neblett, 2017). As such, ethnic identity, in this study, is explored among PoC as a possible protection from racial microaggressions.

Ethnic identity has been explored in the context of racism as an influencing factor for PoC (Bracey et al., 2004; Lou et al., 2022). Research suggests that individuals who have positive attitudes about their group membership report other positive outcomes (Jones and Neblett, 2017). For example, positive ethnic identity attitudes have been linked to higher self-esteem for Black and African Americans (Bracey et al., 2004). Lou et al. (2022) examined the Chinese Canadian experience and tested ethnic identity as a protective factor for the effect of personal and group discrimination on well-being. Findings indicated that Chinese participants who endorsed a strong ethnic identity experienced less adversity when navigating discrimination. In a review on ethnic identity and racism, Jones and Neblett (2017) found strong support for ethnic identity as a moderator for racism and psychological outcomes. The findings around ethnic identity being a “protective” factor, however, are not entirely consistent and can differ across communities of color. Even though there is relatively less evidence for this claim, some studies have found that strong ethnic identity was associated with greater perceived discrimination and negative psychological outcomes (Bair and Steele, 2010; Lee and Ahn, 2013). A meta-analysis across 26 studies on Black Americans showed that greater perceived racial discrimination was associated with strong ethnic identity. Moreover, these effects were linked to self-blame processes related with greater psychological distress (Lee and Ahn, 2013). However, this same meta-analysis found that racial identity (e.g., Afrocentricity) was a protective factor. Psychometric experts have also suggested that ethnic identity affects racism-related experiences differently. Phinney (1992) developed the Multigroup Ethnic Identity Measure (MEIM), categorizing ethnic identity into two developmental stages: (1) search and (2) commitment. The search factor of MEIM has ethnic identity as an explorative phase (e.g., how much does one know about their ethnicity). The second factor on commitment measures how much one feels connected to their ethnic group. The mixed findings around ethnic identity and the association with racism-related experiences raise questions about how protective (or not) ethnic identity is for PoC. *Is ethnic identity a protective factor for PoC when experiencing racial microaggressions? Does ethnic identity protect PoC from blaming themselves when experiencing racism? If so, when does such buffering effect take place?* Previous findings overall suggest that the endorsement of ethnic identity can benefit PoC in some cases. For this study, we were curious about the relationship between ethnic identity and a more hidden type of racism—racial microaggressions. We tested the effectiveness of ethnic identity as a moderator in the relationship between racial microaggressions and self-blame coping strategies for discrimination.

Resilience is another positive attribute often linked to marginalized groups. This is the ability to recuperate from challenging or stressful situations (see Cabrera Martinez et al., 2022, for a review). Like ethnic identity, resilience is sometimes referred to as another protective factor for well-being when experiencing racism (Cabrera Martinez et al., 2022). Resilience is often associated with positive mental health outcomes in the context of oppression (Siriwardhana et al., 2014). Vincent et al. (2020) examined resilience among young Black men who have sex with men, and their findings indicated that resilience was a key protective factor for depression. Findings further indicated that resilience played a critical role in the beneficial effects of peer social support. Based on past evidence, some researchers have highlighted the importance of building and promoting resilience among marginalized youth (Carranza, 2007; Evans and Pinnock, 2007; Bodkin-Andrews et al., 2013). Other scholars have questioned, however, whether the full endorsement of resilience, without considering other factors (e.g., systemic and institutional factors), can be harmful to marginalized groups (Aguilera and Barrita, 2021; Barrita and Wong-Padoongpatt, 2021). Some scholars have called for a *revisit* of the ways in which resilience is defined in the context of oppression (Sims-Schouten and Gilbert, 2022). Sims-Schouten and Gilbert (2022) argue that the reconceptualization of resilience should include how this “trait” is a byproduct or result of continuous and historical oppression. Thus, the question remains whether resilience influences the way PoC experiences racism. Brown and Tylka (2011) found that Black young adults’ resilience was negatively related to racial discrimination only when lower racial socialization was experienced. Moreover, resilience was no longer a significant moderator; and therefore, did not buffer the effect when racial socialization was high. Similar to ethnic identity, these mixed findings leave questions around the protective nature of resilience. While resilience has been explored as a moderator in relation to blatant or systemic racism and well-being among PoC (see Cabrera-Martinez for a review), to our knowledge, resilience has not been tested as a moderator for everyday racial microaggressions in relation to self-blame coping strategies. Thus, this current study examined whether self-reported resilience buffered the relation between self-blame coping strategies and psychological distress.

**Figure 1:**
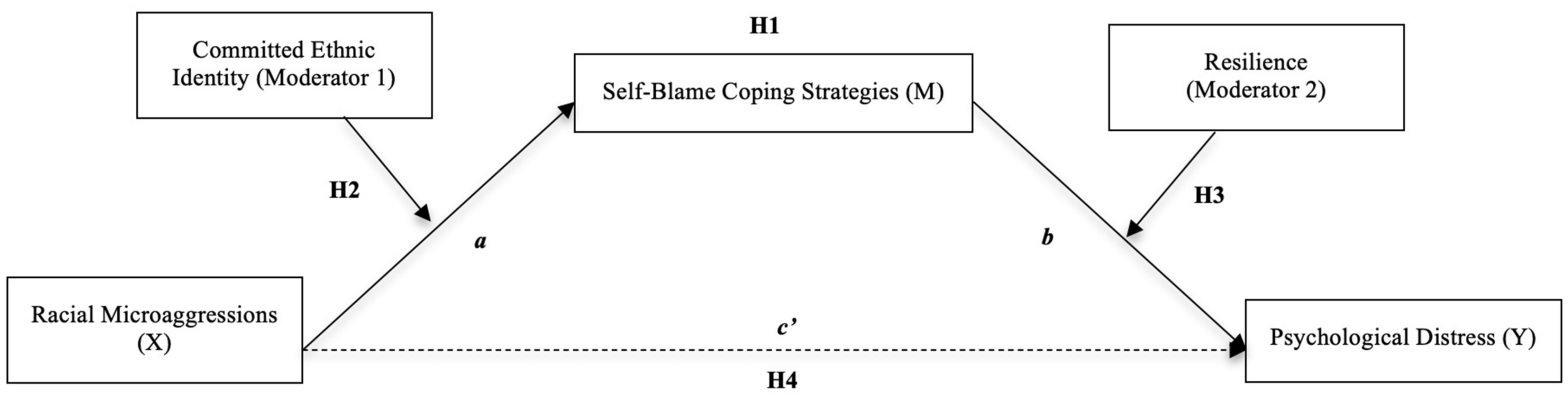
Conceptual moderating effects of committed ethnic identity and resilience on mediated relation of racial microaggressions and psychological distress through internalization.

Our study explored the relationship between racial microaggressions, psychological distress, and self-blame coping for discrimination. Secondly, we tested the internal processes of resilience and ethnic identity as possible protective factors from racial microaggressions. This study is among the few that have examined the influence of resilience and ethnic identity as internal processes within the framework of microaggressions. Specifically, we used a series of mediation, moderation, and conditional analyses (see Figure 1) to comprehensively examine the mechanisms and interactions of the impact of racial microaggressions on psychological distress.

## Current study

Our study unpacked the possible factors associated with racial microaggressions given the increment of racial tension in the U.S. in the last decade (Pew Research Center, 2019). In 2015, microaggressions was named the word of the year and according to a large-scale study on racism, PoC reported experiencing racial microaggressions consistently (American Psychological Association, 2016). There have been recent recommendations to explore power, privilege, and protective factors when unpacking discrimination experiences (Sims-Schouten and Gilbert, 2022; Suyemoto et al., 2022). The current study assessed how self-blaming coping strategies can explain the link between racial microaggressions and psychological distress. Furthermore, we tested if ethnic identity and resilience can be protective factors that moderate these relations. Thus, we tested the following hypotheses.

*H1*: The relationship between racial microaggressions and psychological distress will be mediated by self-blame (Mediation model).

*H2*: The relationship between racial microaggressions and self-blame will be moderated by the level of committed ethnic identity, such as that lower levels of committed ethnic identity will be associated with higher levels of self-blame when experiencing racial microaggressions (Moderator 1).

*H3*: The relationship between self-blame and psychological distress will be moderated by the level of resilience, such as that lower levels of resilience will be associated with higher levels of psychological distress when self-blame coping (Moderator 2).

*H4*: Conditional hypothesis. If H1-H3 are supported, we hypothesize that both committed ethnic identity and resilience will moderate the mediated relation between racial microaggressions, self-blame, and psychological distress. (Double moderated mediation model). See Figure 1 for the proposed model.

## Materials and methods

### Participants

We collected data during the Fall of 2020 using a cross-sectional online survey. We recruited a convenience sample from two sources: (1) college students from a diverse southwest university in the U.S. who participated for class credit, and (2) voluntary participants from social media (e.g., Facebook, Twitter) who accessed the survey through an advertised flyer without compensation. Inclusion criteria for the study included: (a) be 18 years or older, (b) be fluent in English, (c) currently reside within the U.S., and (d) identify as a member of a racial or ethnic minoritized group. A sample of 702 participants was initially collected for this study, with 48% of participants (*n* = 337) recruited from the southwest institution. Three participants were removed for not passing attention checks (e.g., asking to mark a specific response for an additional item on each quantitative measure randomly placed). Additionally, three more participants were removed for being flagged as significant outliers across our main variables in a linear regression model (racial microaggressions, self-blame, psychological distress, ethnic identity, and resilience). Significant outliers were those that scored outside of recommended cut-offs for two or more of the three recommended outlier checks (Cook’s, Mahalanobis, and Leverage distance values). Thus, a final sample of 696 participants was kept for this study. The demographic information for our sample is displayed in Table 1.

**Table 1:** Sociodemographic characteristics of participants.

Baseline characteristic		
	*n*	%
Gender		
Ciswomen	510	73.3
Cismen	172	24.7
Transgender	3	0.4
Non-conforming	4	0.6
Non-binary	4	0.6
Other	3	0.4
Race/Ethnicity		
Black or African American (non-Latinx)	115	16.5
Southeast Asian	142	20.4
East Asian	102	14.7
Black, Afro, or Caribbean Latinx	83	11.9
Indigenous Latinx	156	22.4
White Latinx	98	14.1
Immigrant generation		
1^st^ Generation	32	4.6
1.5 Generation	82	11.8
2^nd^ Generation	367	52.7
3^rd^ Generation	89	12.8
4^th^ or above generation	126	18.1
Sexual Identity		
Heterosexual	537	77.2
Gay or Lesbian	42	6.0
Bisexual	68	9.8
Queer	9	1.3
Pansexual	14	2.0
Asexual	6	0.9
Prefer not to disclose	20	2.9

*M_age_* = 22.26 years old (*SD* = 3.84). MSES = 5.50 (*SD* = 1.60) using a self-rated scale 1 to 10 for socioeconomical status.

### Procedures

This study was exempted by a university institutional review board (IRB# UNLV-1572714) and conducted based on federal and university regulations. Each participant was informed of the purpose of the study at the beginning of the survey and was asked for consent to participate. After consent was obtained, participants were asked to answer various quantitative measures, provide demographic information, and received psychological resources during the final debriefing section.

### Measures

#### Racial and ethnic microaggressions

The Racial and Ethnic Microaggressions Scale (REMS; Nadal, 2011) is a 45-item instrument that measures the frequency of racial and ethnic microaggressions experienced in the last 6 months. Participants are asked to report how often they have experienced scenarios such as “Someone assumed I was not intelligent because of my race,” using a 6-point Likert scale ranging from 0 (*I did not experience this event*) to 5 (*I experienced this event five or more times*). Higher scores indicate higher levels of racial and ethnic microaggressions experiences. REMS has been consistently used to assess everyday discrimination experiences among PoC, showing consistent and high reliability (Nadal, 2011; Barrita et al., 2023a). For this study, REMS showed strong reliability with α = 0.91.

#### Self-blame coping

The Coping with Discrimination/Internalization subscale (CDS-I; Wei et al., 2010) is a 5-item measure that assesses participants’ self-blame as a coping strategy when experiencing discrimination. Participants are asked to report their level of agreeableness to items such as “I wonder if I did something to provoke this incident,” using a 6-point Likert scale ranging from 0 (*never like me*) to 5 (*always like me*). Higher scores indicate higher levels of self-blaming experiences with discrimination. CDC-I has shown strong reliability in previous studies exploring racial microaggressions (Wei et al., 2010; Barrita et al., 2023b). For this study, CDS-I showed a reliability of α = 0.83, and full CDS produced a Cronbach’s alpha of α = 0.84.

#### Psychological distress

The Depression, Anxiety, and Stress Scale (DASS-21; Antony et al., 1998) is a 21-item measure that assesses symptoms of depression, anxiety, and stress (seven items for each category). Participants are asked to report their level of agreeableness to items such as “I felt that I had nothing to look forward to,” using a 4-point Likert scale ranging from 0 (*did not apply to me*) to 4 (*applied to me most of the time*). Higher scores on this scale suggest more evidence for symptoms of psychological distress. DASS has shown strong reliability in previous studies exploring the relationship between racial microaggressions and psychological distress (Wong-Padoongpatt et al., 2022a, b, c). For this study, DASS produced a Cronbach’s alpha of α = 0.91.

#### Ethnic identity

The Multiethnic Ethnic Identity Measure Affirmation Subscale (MEIM-A; Phinney, 1992) is a 7-item measure that assesses participants’ sense of affirmation, belonging, and commitment to their ethnic identity. For this study, we intentionally tested only the committed version using the MEIM Affirmed subscale (Phinney, 1992), as previous findings indicated that other types of ethnic identity (i.e., searching or in development) provide less protection (Jones and Neblett, 2017). Participants are asked to report their level of agreeableness to items such as “I have a clear sense of my ethnic background and what it means,” using a 5-point Likert scale ranging from 0 (*strongly disagree*) to 4 (*strongly agree*). Higher scores suggest higher levels of commitment to one’s ethnic identity. MEIM has been used in previous studies connected to racial discrimination showing strong reliability (Lee and Ahn, 2013). For this study, MEIM-A produced a Cronbach’s alpha of α = 0.88.

#### Resilience

The Brief Resilience Scale (BRS; Smith et al., 2008) is a 6-item scale that assesses the perceived ability to bounce back. Participants are asked to report to which extent they agree to items such as “It does not take me long to recover from a stressful event,” using a 5-point Likert scale ranging from 1 (*strongly disagree*) to 5 (*strongly agree*). Higher scores suggest higher levels of resilience. BRS has shown strong reliability in previous studies using PoC samples (Barrita, 2021; Grooms et al., 2021). For this study, BRS produced a Cronbach’s alpha of α = 0.90.

#### Demographics

Participants provided demographic information about their racial and ethnic identity, socioeconomic status (SES), age, gender identity, and sexual orientation. Additionally, the immigrant generation was assessed based on the following categories: (a) *1^st^ generation*: you immigrated to the U.S. after age 12, (b) *1.*5 *generation:* you immigrated to the U.S. at or before age 12, (c) *2^nd^ generation:* you were born in the U.S., and at least one of your parents immigrated to the U.S., (d) *3^rd^ generation:* you and your parents were born in the U.S., and at least one of your grandparents immigrated to the U.S., (e) *4^th^ generation:* you, your parents and your grandparents were all born in the U.S. (see Table 1 for full demographic info).

### Statistical analysis

We used SPSS 28.0 (SPSS, Inc., Chicago, IL) to analyze the data for this study. The analysis plan included a preliminary analysis of assumptions and significant outliers and a moderated mediation analysis using Hayes’ (2012) PROCESS model 21 to test our hypotheses. To test all four of our hypotheses, we conducted a moderated mediation analysis using 5,000 bootstrap samples based on Hayes’ (2012) PROCESS macro-Model 21 (two moderators within mediation) exploring: a) if the relationship between racial microaggressions (IV) and psychological distress (DV) is explained by self-blame (Mediator), b) if committed ethnic identity (moderator 1) influences the relation between racial microaggression and self-blame (*path a*), c) if resilience (moderator 2) influences the effect of self-blame on psychological distress (*path b*), and d) if overall, the link between racial microaggressions and psychological distress can be explained by self-blame and influenced by both committed ethnic identity and resilience (moderated mediation). To test moderation effects, we assessed specific regions of each interaction based on standard deviation levels (i.e., 1 SD below mean, mean, and 1 SD above mean). We used simple slope analyses (Johnson and Fay, 1950) as this technique is considered superior to locating regions of significance for an interaction (Hayes, 2012) and has been used in similar studies focused on racial microaggressions using a moderated mediation model (Barrita et al., 2023b).

## Results

Our preliminary analysis found three significant outliers, which were removed prior to the main analysis for our five main continuous variables (racial microaggressions, self-blame, psychological distress, committed ethnic identity, and resilience). Similarly, we checked for homoscedasticity, independence errors, and multicollinearity, finding no concerns to conducting our main analyses. We checked for covariates among our categorical variables. Results from one-way ANOVA’s showed no significant difference around sexual orientation or immigrant generation for our five main continuous variables. Around gender, 11 participants (2%) self-identified as gender expansive, which, compared to the other two groups’ sample sizes (ciswomen and cismen), made it impossible to compare. We conducted various t-tests to compare cisgender groups for our main variables. Results for committed ethnic identity between cismen (*M* = 21.98, *SD* = 4.85) and ciswomen (*M* = 23.63, *SD* = 4.05) indicated significant differences *t*(684) = −4.55, *p* < 0.001, *d* = 0.39; thus, gender was considered a covariate. We conducted a Pearson’s bivariate correlation analysis for other continuous variables such as age or SES (see Table 2). Results suggested that neither SES nor age were significantly associated with our main continuous variables. Gender was the only covariate controlled for during our main analysis.

**Table 2:** Bivariate correlations.

Measure	Mean	*SD*	1	2	3	4	5	6	7
Racial Microaggressions	16.54	9.55	—						
Psychological distress	40.28	11.10	0.239^**^	—					
Self-blame	14.09	5.42	0.127^**^	0.323^**^	—				
Affirmed racial identity	23.21	4.32	0.098^**^	−0.161^**^	−0.133^**^	—			
Resilience	19.31	4.66	−0.069	−0.438^**^	−0.209^**^	0.101^*^	—		
Age	22.26	3.84	0.193	0.135	−0.141	−0.057	0.218	—	
SES	5.50	1.60	−0.070	−0.153	0.052	0.136	0.146	0.222	—

*N* = 696.

* *p* < 0.05;

** *p* < 0.01.

### H1: Mediation

Results from our model highlighted that racial microaggressions were a significant and positive predictor of self-blame *B* = 0.375, *SE* = 0.114, *p* < 0.001, 95% CI [0.152, 0.598], self-blame was a significant predictor of psychological distress *B* = 0.697, *SE* = 0.253, *p* < 0.001, 95% CI [0.201, 1.193]. Evidence of partial mediation was found as racial microaggressions significantly predicted a change in psychological distress after controlling for self-blaming *B* = 0.214, *SE* = 0.038, *p* < 0.001, 95% CI [0.140, 0.288] (direct effect), while a significant coefficient was obtained for the model *B* = 0.002, *SE* = 0.001, *p* < 0.001, 95% CI [0.001, 0.003] (indirect effect). Thus, H1 was partially supported.

### H2: Committed ethnic identity (moderator 1)

**Figure 2:**
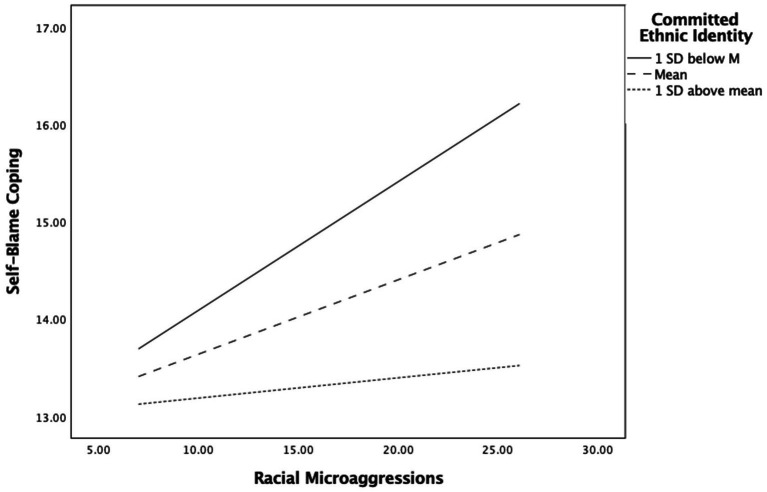
Moderating influence of committed ethnic identity on the association of racial microaggressions and Self-Blame Coping strategies for discrimination.

Using the same Hayes’ macro-Model 21 (Hayes, 2012), we tested if committed ethnic identity moderated path *a* (racial microaggressions and self-blame) in our mediation model. Results suggested that committed ethnic identity was a significant negative predictor of self-blame *B* = −0.224, *SE* = 0.092, *p* < 0.05, 95% CI [−0.246, −0.056]. The interaction of committed ethnic identity and racial microaggressions also significantly predicted a change in self-blame *B* = −0.013, *SE* = 0.005, *p* < 0.01, 95% CI [−0.022, −0.003]. Specifically, there was a positive relation between racial microaggressions and self-blame, which was moderated by committed ethnic identity, where the higher this was, the lower levels of self-blame were reported. This path model accounted for 4.72% of the variance, with the interaction accounting itself for 1% unique variance, *F*(1,692) = 6.96, *p* <. 01. Figure 2 visually depicts a slope analysis of the interaction, which shows that racial microaggressions were significantly predicting more self-blame only when committed ethnic identity was 1 *SD* below the mean (Effect = 0.132, *p* < 0.001, 95% CI [0.075, 0.189] and at its mean (Effect = 0.076, *p* < 0.001, 95% CI [0.034, 0.118]. But when committed ethnic identity was 1 *SD* above the mean, racial microaggressions were no longer a significant predictor of self-blame (Effect = 0.021, *p* = 0.501, 95% CI [−0.040, 0.081], suggesting that when PoC participants reported high levels of belongingness and commitment to their ethnic identity, they did not internalize racial microaggression. Thus, committed ethnic identity can serve as a protective factor against racial microaggressions and moderated its association with self-blame, supporting our H2.

### H3: Resilience (moderator 2)

**Figure 3:**
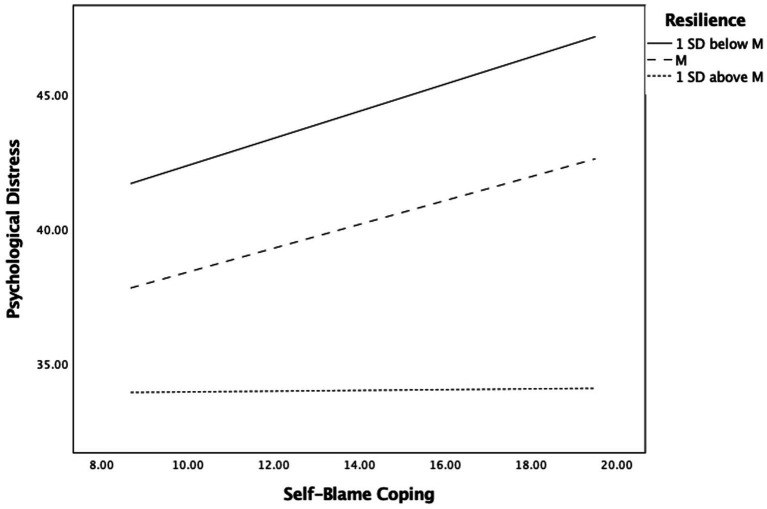
Moderating influence of resilience on the association Self-Blame Coping strategies for discrimination and psychological distress.

Next, we tested if resilience moderated path *b* (self-blame coping and psychological distress) in our mediation model. Results suggested that resilience was a significant negative predictor of psychological distress *B* = −0.721, *SE* = 0.198, *p* < 0.001, 95% CI [−1.109, −0.332]. The interaction of resilience and self-blame also significantly predicted a change in psychological distress *B* = −0.113, *SE* = 0.013, *p* < 0.05, 95% CI [−0.203, −0.038]. Specifically, there was a positive relation between self-blame and psychological distress which was moderated by resilience, such as the higher resilience was, the lower psychological distress levels were reported. Path *b* in our model accounted for 29.3% of the variance, with the interaction accounting itself for 3.1% unique variance [*F*(1,691) = 2.01, *p* < 0.05]. Figure 3 shows a slope analysis of the interaction where self-blame significantly predicted more psychological distress only when resilience was 1 *SD* below the mean (Effect = 0.065, *p* < 0.001, 95% CI [0.015, 0.103] and at its mean (Effect = 0.060, *p* < 0.001, 95% CI [0.012, 0.089]. But when resilience was 1 *SD* above the mean, self-blame was no longer a significant predictor of psychological distress (Effect = 0.005, *p* = 0.324, 95% CI [−0.008, 0.023]. Thus, resilience can serve as a protective for mental health when engaging in self-blaming coping strategies associated with racial microaggressions, therefore, supporting our H3.

### H4: A moderated mediation

Overall, our model supported our H4 (see Table 3), as self-blame helped explain the association between racial microaggressions and psychological distress. Furthermore, both committed ethnic identity and resilience moderated this mediating effect (Index = 0.002, *BootSE* = 0.001, 95% BootCI [0.001, 0.003]. Table 4 describes the specific indirect effect of the model based on three levels (1SD below mean, at mean, and 1SD above mean) for each of our moderators.

**Table 3:** Moderated mediation model characteristics for predictors on self-blaming coping strategies (mediator) and psychological distress (dependent variable).

	Internalizing coping strategy			Psychological distress		
Predictor	B	SE	95% CI	B	SE	95% CI
Racial microaggressions (X)	0.375^***^	0.114	0.152; 0.598	0.214^***^	0.038	0.140: 0.288
Self-blaming (M)				0.691^**^	0.253	0.201: 1.192
Affirmed ethnic ID (W)	−0.224^*^	0.092	−0.205: −0.056			
X × W**[H2]**	−0.013^**^	0.005	−0.022: −0.003			
Resilience (V)				−0.721^***^	0.198	−1.109: −0.333
M × V**[H3]**				−0.113^*^	0.013	−0.203: −0.037
X > W > M > V**[H4]**				0.002^*^	0.001	0.001: 0.003
Sex	−0.720	0.451	−1.606: 0.165	−1.170	0.003	−0.001: 0.645
Model R^2^	**0.047, *F*(3,691) = 11.41 *p* < 0.001**			**0.283, *F*(4,691) = 68.16, *p* < 0.0001**		
Interaction Δ^2^	**0.010, *F*(1,692) = 6.96, *p* < 0.01**			**0.031, *F*(2,691) = 12.44, *p* < 0.05**		

*N* = 696.

* *p* < 0.05;

** *p* < 0.01;

*** *p* < 0.001.

Bold values are statistically significant.

**Table 4:** Conditional indirect effects of racial microaggressions on psychological distress through self-blame accounting for affirmed ethnic identity and resilience as moderators.

Affirmed ethnic identity	Resilience	Indirect effect or index	BootSE	Boot 95% CI
Low	Low	0.067	0.023	**0.027: 0.116**
Low	Average	0.059	0.018	**0.027: 0.096**
Low	High	0.051	0.017	−0.020: 0.088
Average	Low	0.039	0.014	**0.014: 0.070**
Average	Average	0.034	0.011	**0.014: 0.057**
Average	High	0.029	0.010	−0.011: 0.052
High	Low	0.011	0.007	**0.010: 0,044**
High	Average	0.009	0.014	−0.019: 0.037
High	High	0.008	0.012	−0.017: 0.032

*N* = 696. Bold values are statistically significant.

### Post-hoc analyses

As part of the review process, additional comparative analysis across race and ethnicity was suggested to assess if there were significant differences across the subgroups within our sample. Given our findings about the direct and indirect effects of the mediation model portion of our study, we tested race/ethnicity (see subgroups in Table 1) using Hayes’ (2012) PROCESS Model 59 moderated mediation with 5,000 bootstrapped samples. This model can be used to test one single categorical moderator (race/ethnicity) across all three paths of the mediation. Results for this model suggested there was no evidence of moderated mediation for any group, as zero was found between *boot* confidence intervals (95% bias-corrected) for the difference of conditional indirect effects suggesting no significant coefficients for any racial/ethnic group. However, these results should be conservatively interpreted, given that sample sizes for subgroups might have been too small therefore underpowering this analysis. Furthermore, the literature does suggest that there are specific differences in our main variables based on race and ethnicity (Ponterotto and Park-Taylor, 2007; Bailey et al., 2019; Aguilera and Barrita, 2021; Cabrera Martinez et al., 2022).

## Discussion and implication

The findings supported all four of the study’s hypotheses. First, we wanted to know if self-blame explained the link between racial microaggressions and psychological distress. Results from our mediation model supported H1 and suggested that those who experienced more racial microaggressions were more likely to endorse self-blame coping for discrimination. Furthermore, those who endorsed high levels of self-blame coping also exhibited more psychological distress. These results are consistent with previous findings exploring other forms of racism (e.g., systematic, institutional) where PoC engaged in self-blaming coping behaviors that predicted psychological distress (David et al., 2019). Our findings are also consistent with IOT (David, 2013), as PoC in our sample not only reported experiencing racial microaggressions but also reported blaming themselves for discriminatory experiences. Our findings suggest that self-blame coping can explain the psychological impact of racial microaggressions on stress, depression, and anxiety. These results can inform current clinicians and practitioners assessing race-related stressors about specific factors, such as self-blaming behaviors associated with experiencing racial oppression.

Results for our H2 confirmed that low and average levels of committed ethnic identity moderated the relation between racial microaggressions and self-blame. Only high levels of committed ethnic identity disrupted such relations. Results for our first moderator analysis suggested that committed ethnic identity, which previous studies have described as a key protective factor against racism (Jones and Neblett, 2017), did not serve as such for those with low or average levels of committed ethnic identity. Only those reporting ethnic identity one standard deviation above its mean were the only participants that no longer reported significant levels of self-blame when experiencing racial microaggressions. These results support previous critiques (Bair and Steele, 2010; Lee and Ahn, 2013) that challenge arguments about ethnic identity protective traits and minimize the impact systemic and individual oppression has over time on PoC and its influence on ethnic identity protective traits. Our findings suggest that PoC can endorse and commit to their ethnicity and still blame themselves for hostile messages. It is also possible that, given that racial microaggressions are consistent stressors (Sue et al., 2007) taking place in one’s microsystem, it might be hard to quickly find positive coping strategies (e.g., externalization and resistance). Our results showed that self-blame was still reported for many participants in our sample who had low or average levels of committed ethnic identity. Therefore, the issue remains on systemic and individual oppression and not on one’s sense of belonging to their ethnic group.

Our findings around H3 for resilience found similar results as for ethnic identity. At low and average levels, resilience moderated the relationship between self-blame coping and psychological distress. However, the relationship between self-blame coping and psychological distress was no longer significant when resilience was one standard deviation above the mean. These findings challenge consistent descriptions of PoC’s resilience as a necessary or effective factor that shields from oppression (Evans and Pinnock, 2007). For our sample, the *foretold* protection of resilience was only effective at very high levels, suggesting that such a protective effect is the exception and not the rule for such a trait. This finding does support critiques that question the absolute positivity of resilience (Aguilera and Barrita, 2021; Barrita and Wong-Padoongpatt, 2021; Sims-Schouten and Gilbert, 2022). Our results around the effectiveness of resilience on psychological well-being can also be explained by the detrimental effects of internalized racism (e.g., self-blame) on PoC (see David et al., 2019 for a review). Perhaps resilience, which can be protective in some cases (Evans and Pinnock, 2007), did not moderate in our model because it is harder to psychologically recuperate once one has blamed themselves for these experiences. These findings provide additional evidence to the current debate around self-reported resilience and its protective nature.

Finally, supportive evidence for our moderated mediation model (H4) was found, and results suggested that the relationship between racial microaggressions and psychological distress was partially mediated by self-blame, which was moderated by both committed ethnic identity and resilience at low and average levels. Specifically, indirect effects for both moderators showed how the psychological impact linked to self-blaming differed based on moderating levels (e.g., low, average, and high) for ethnic identity and resilience. Furthermore, this model also showed how both moderators at high levels no longer produced a significant relation for our main variables. Our findings add evidence to current counter-discussions on the effectiveness or protective value both ethnic identity and resilience might have when facing oppression. Our study explored various factors previously studied for systemic racism in relation to everyday racial microaggressions. Therefore, our findings can also inform how cumulative effects from harder-to-identify aggressions can impact PoC’s well-being. Larger implications from our study include informative evidence to help clinicians improve their assessment of race-related stressors or consider other factors, such as PoC’s self-blaming coping strategies connected to racism. Furthermore, our findings challenge the assumptions that PoC’s ethnic identity or resilience can serve as absolute protection from racism. Our study meticulously unpacked these traits and findings showed that ethnic identity and resilience only protected some – and not the majority – putting back the responsibility to liberate PoC on the oppressor and the mechanisms upholding the racialized system.

## Limitations and conclusion

Our study carries important limitations that can guide future research on the impact of racial microaggressions. First, our cross-sectional study limited participants’ responses to one-period time and asked participants to recall racial-related experiences that cannot always be identified as aggressions (Sue et al., 2007). Our sample was also collected during a global pandemic that sparked higher racial tension for some groups in the U.S., including Asian Americans (Wong-Padoongpatt et al., 2022a,b,c), and in the same year when the world saw a rise of social movements and protests demanding justice for George Floyd and other Black lives from police brutality (Reny and Newman, 2021). As such, the timing of our data collection might have influenced the psychological distress and racial awareness among our participants. Causal relations or distinctions between which factors (e.g., resilience, ethnic identity, and psychological distress) occurred first cannot be claimed given our methodological approach. The evidence of ethnic identity serving as an absolute protective factor is inconsistent, and it is possible that other identities can also serve as protective factors (e.g., racial identity). Furthermore, cultural and developmental factors, such as differences in participants’ direct environments (e.g., racially homogenous or heterogenous), were not assessed, which could have influenced experiences with racial microaggressions or participants’ ethnic identity development (Helms, 1990). Thus, future studies exploring the influence of ethnic identity and racism should consider expanding on these factors. Similarly, our study assessed resilience as defined by the BRS (Smith et al., 2008), which measures only internal-individual self-reported levels of resilience and no other types of, such as community-resilience or spiritual-resilience also reported by minoritized groups (Aguilera and Barrita, 2021). It is possible that external forms of support, such as community, might produce different levels and effects of protection that can be defined as resilience too. Future studies on the effects of racism may consider experimental or longitudinal approaches that can further unpack different types and levels of resilience as protective factors. Moreover, the effect sizes for our significant findings were small. Thus, some of these changes in psychological distress are likely imperceivable by the participants. Finally, future research on the liberation of PoC and other marginalized communities might benefit from intersectional approaches that test not only individual levels of microaggressions but also systemic and institutional levels.

Despite the limitations of this study, our results produced evidence that can inform researchers and clinicians about the effects of racial microaggressions and relevant factors. Our study explored multiple relations using a combined moderated mediation model, which tested two factors constantly described as protective in the relation between racism and well-being. Our findings suggest that ethnic identity and resilience can influence the impact PoC experience from racism. But more importantly, our study brings awareness to how PoC are still considerably impacted by racial microaggressions despite these traits. Thus, PoC can continue to commit strongly to their ethnic identity and be resilient, yet still, be significantly impacted by racial microaggressions. Thus, ethnic identity and resilience are important individual-level factors, but the endorsement of these traits should be discussed within a larger sociopolitical context. PoC in the U.S. have needed to develop traits and strategies to cope with and recuperate from decades of racism. Thus, true changes toward a more equitable and just world demand that those in positions of power and privilege match the resilient spirit of the oppressed and engage actively against oppression.

## Data availability statement

The raw data supporting the conclusions of this article will be made available by the authors, without undue reservation.

## Ethics statement

The studies involving human participants were reviewed and approved by UNLV. The patients/participants provided their written informed consent to participate in this study.

## Author contributions

All authors listed have made a substantial, direct, and intellectual contribution to the work and approved it for publication.

## Conflict of interest

The authors declare that the research was conducted in the absence of any commercial or financial relationships that could be construed as a potential conflict of interest.

## Publisher’s note

All claims expressed in this article are solely those of the authors and do not necessarily represent those of their affiliated organizations, or those of the publisher, the editors and the reviewers. Any product that may be evaluated in this article, or claim that may be made by its manufacturer, is not guaranteed or endorsed by the publisher.
